# Network-based Phenome-Genome Association Prediction by Bi-Random Walk

**DOI:** 10.1371/journal.pone.0125138

**Published:** 2015-05-01

**Authors:** MaoQiang Xie, YingJie Xu, YaoGong Zhang, TaeHyun Hwang, Rui Kuang

**Affiliations:** 1 College of Software, Nankai University, Tianjin, China; 2 Department of Clinical Science, University of Texas Southwestern Medical Center, Dallas, TX, USA; 3 Department of Computer Science and Engineering, University of Minnesota Twin Cities, Minneapolis, MN, USA; University of Padova, ITALY

## Abstract

**Motivation:**

The availability of ontologies and systematic documentations of phenotypes and their genetic associations has enabled large-scale network-based global analyses of the association between the complete collection of phenotypes (phenome) and genes. To provide a fundamental understanding of how the network information is relevant to phenotype-gene associations, we analyze the circular bigraphs (CBGs) in OMIM human disease phenotype-gene association network and MGI mouse phentoype-gene association network, and introduce a bi-random walk (BiRW) algorithm to capture the CBG patterns in the networks for unveiling human and mouse phenome-genome association. BiRW performs separate random walk simultaneously on gene interaction network and phenotype similarity network to explore gene paths and phenotype paths in CBGs of different sizes to summarize their associations as predictions.

**Results:**

The analysis of both OMIM and MGI associations revealed that majority of the phenotype-gene associations are covered by CBG patterns of small path lengths, and there is a clear correlation between the CBG coverage and the predictability of the phenotype-gene associations. In the experiments on recovering known associations in cross-validations on human disease phenotypes and mouse phenotypes, BiRW effectively improved prediction performance over the compared methods. The constructed global human disease phenome-genome association map also revealed interesting new predictions and phenotype-gene modules by disease classes.

## Introduction

**Fig 1:**
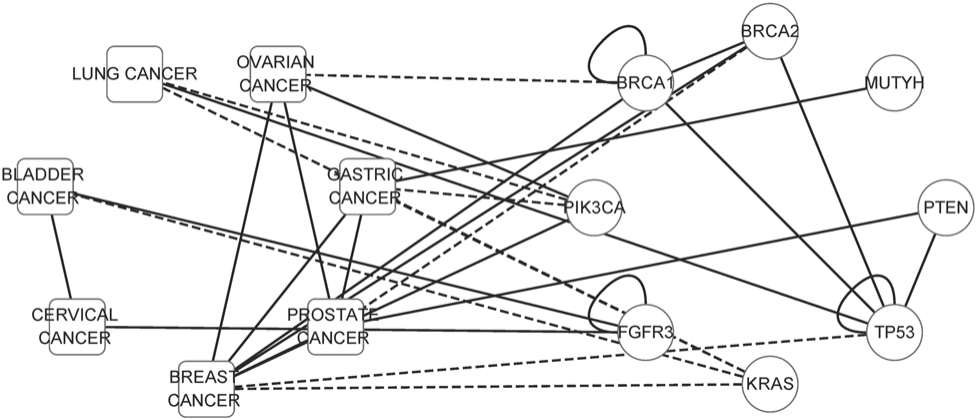
Predicting missing associations in disease phenotype-gene association network. This real subnetwork includes seven cancer phenotypes and eight disease genes. Similar phenotypes and interacting genes are connected. The solid lines represent the OMIM association known before May, 2007. The dash lines represent new OMIM associations added after May, 2007.

In the past decade, large-scale efforts have been put into establishing ontologies and documentations to describe the full collection of phenotypes (called phenome). The generated large phenotype databases and ontologies categorize phenotypes in many species [1–3] and human genetic diseases [4]. The global analysis of the collection of phenotypes in a database or an oncology and the known phenotype-gene relations now provides a strategy to predict a list of candidate genes based on the knowledge of already determined phenotype-gene associations such as those in Mouse Genome Informatics (MGI) [5] and Online Mendelian Inheritance in Man (OMIM) [4], a database of human genes and genetic disorders. Complimentary to high-throughput genomic profiling approaches, this knowledge-based strategy takes the advantage of the availability of large phenotypic and molecular networks such as human disease phenotype network [6], human protein-protein interaction network [7, 8] or functional linkage network [9]. The problem of predicting phenotype-gene associations in a real phenotype-gene association subnetwork is illustrated in Fig 1. Based on the assumption that the relations among the genes and among the diseases in the networks are predictive of their associations, many network-based approaches have been proposed to utilize the disease modules and gene modules in the networks to prioritize disease genes for a disease phenotype [9–19], to find related disease phenotypes for a gene set [20] or to detect disease-gene modules [21]. A more recent method further explored protein complex relation in the networks to improve gene prioritization [19]. Despite of the impressive results in the studies, few attempts have been made to explain the network-based prediction approaches by graph patterns.

We postulate that the relation among phenotype-gene associations can be characterized by circular bigraph patterns (CBGs). Based on the observation of high frequency of CBGs in MGI and OMIM associations, we apply a bi-random walk algorithm (BiRW) [22] to capture the CBG patterns in the networks for unveiling the association between the complete collection of phenotypes and genes (phenome-genome association). The key assumption is that the global structure of phenome-genome association can be represented by many overlaying circular bigraphs, i.e. each phenotype-gene association is likely to be paired with some other phenotype-gene association(s) with their phenotypes and genes closely related in the phenotype network and the gene network, respectively. The assumption is supported by the phenotype-gene modules reported in [21] as well as the observation of frequent CBGs in this study. Thus, the reconstruction of the complete phenome-genome association can be achieved by maximizing the number of circular bigraphs balanced with the known associations.

BiRW iteratively adds new associations into the network by bi-random walk to evaluate the number of recovered circular bigraphs with a decay factor penalizing longer paths in the CBG patterns. Note that different from the algorithms for disease gene prioritization [9, 13–16], which rank genes for a particular query phenotype, BiRW is a global approach to reconstruct the missing associations for all the phenotypes simultaneously.

## Methods

A phenotype-gene association network is a heterogeneous network composed of a phenotype network, a gene network and the phenotype-gene associations modeled by a bipartite graph (Fig 1). Let *P*_(*m*×*m*)_, *G*_(*n*×*n*)_ and *A*_(*m*×*n*)_ be the affinity matrix of the phenotype network, the gene network and the association bipartite graph respectively, where *m* is the number of phenotypes and *n* is the number of genes. The objective is to predict the missing associations based on the heterogeneous disease phenotype-gene association network by reconstructing an association matrix *R*_(*m*×*n*)_. The magnitude of each *R*_*ij*_ provides the degree of association between phenotype *i* and gene *j*. In the following, we first introduce the concept of circular bigraphs (CBG) and then the bi-random walk algorithm (BiRW) for learning *R*_(*m*×*n*)_.

### Circular Bigraphs

**Fig 2:**
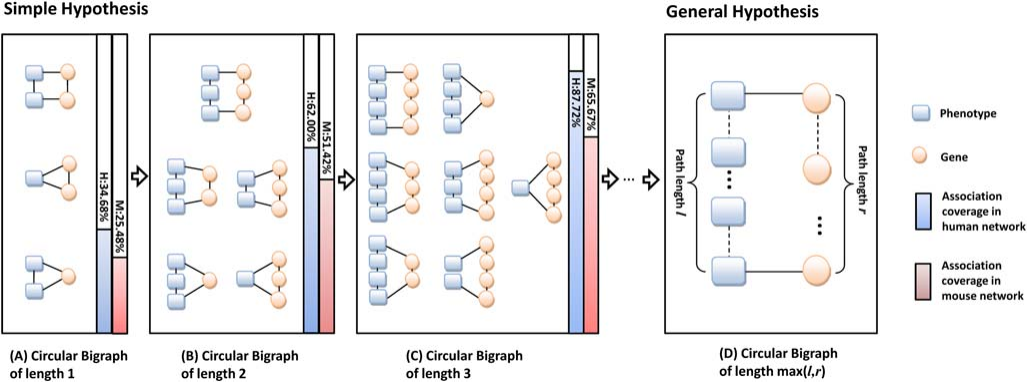
Circular Bigraphs (CBG) in phenome-genome association network. A circular bigraph is composed of a phenotype path and a gene path with two ends connected by phenotype-gene associations. (A), (B) and (C) CBGs with phenotype or gene paths of length 1, 2 and 3. CBGs of length 1 represent the simple hypothesis. (D) The general hypothesis that CBG with phenotype or gene paths of arbitrary length. The blue bar on the right of the figures reports the percentage of OMIM disease phenotype-gene associations that are covered by CBGs up to a certain path length. The red bar reports the percentage in the MGI mouse phenotype-gene associations.

A CBG is defined as a subgraph composed of a phenotype path {*p*_1_, *p*_2_, …, *p*_*m*_} and a gene path {*g*_1_, *g*_2_, …, *g*_*n*_} with two ends connected by two phenotype-gene associations (*p*_1_, *g*_1_) and (*p*_*m*_, *g*_*n*_) (Fig 2). A CBG indicates a vicinity relation between two associations (*p*_1_, *g*_1_) and (*p*_*m*_, *g*_*n*_) by generalizing the relations between *p*_1_ and *p*_*m*_ by their distance in the phenotype network and the relation between *g*_1_ and *g*_*n*_ in the gene network. The smallest CBGs directly represent the simple hypothesis by the existing methods as illustrated in Fig 2A. The triangle with two phenotype nodes and one gene node follows the assumption “similar phenotypes may share the same causal gene”. The triangle with one phenotype node and two gene nodes follows the assumption “causal genes of the same disease phenotype tend to interact”. The rectangle with two phenotype nodes and two gene nodes follows the assumption “genes associated with similar phenotypes tend to interact”. In Fig 2B–2D, we generalize the hypothesis to CBGs of length (*l*, *r*) by exploring the affinity relations captured by the phenotype path and the gene path of longer lengths. We hypothesize that in the unknown complete disease phenome-genome association network, most of the associations tend to be captured by many very small circular bigraphs. Simple calculations of the CBGs in the OMIM phenome-genome association network confirm that, among 1987 OMIM disease phenotype-gene associations, only 34.68% associations are covered by simple hypothesis (Fig 2A), while more than 87% of the known OMIM associations are covered by at least one CBG of path length up to 3 (Fig 2C). CBGs of small path lengths are prevalent in the human disease phenome-genome association network. Some real CBG examples of different path lengths are given in S1 Fig. In the mouse phenome-genome association network, the percentage of associations covered by CBG of length up to 1 or 2 are similarly high with lower coverage by CBG of length 3, which is still significantly higher than the expected coverage in networks with randomized phenotype-gene associations (S11 Table). More detailed results will be discussed in the Section **Results**.

**Fig 3:**
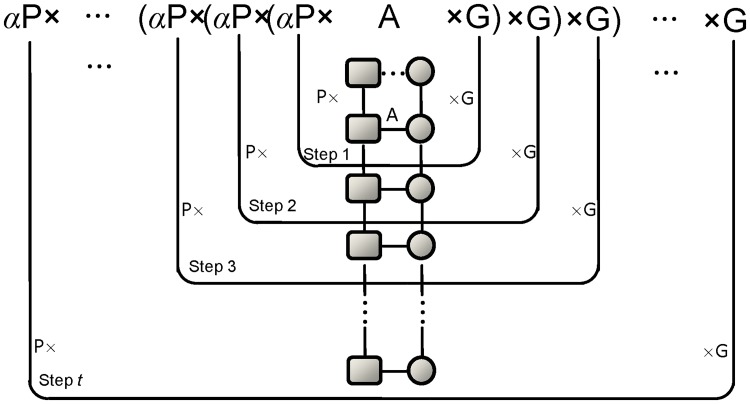
Illustration of bi-random walk. *P* and *G* are the affinity matrices of the phenotype network and the gene network, respectively. *A* is the bipartite graph of the known phenotype-gene association from OMIM. By iteratively extending the phenotype path and the gene path (achieved by multiplying *P* on the left or *G* on the right in each step), the algorithm explores the CBGs weighted by a decay factor *α* ∈ (0,1). The dashed edge indicates a potential association to add into the network. The iterative algorithm finds the number of new CBGs formed by introducing this additional connection. In other words, a potential association is evaluated by its distance to the known associations in the phenotype network and the gene network.

Based on the observation of high CBG frequencies in MGI and OMIM associations, BiRW is specifically designed to capture the CBG patterns in the networks for unveiling phenome-genome association. BiRW aims to explore the CBGs by bi-random walk on both phenotype network and gene network to evaluate potential candidate associations (Fig 3). By iteratively extending the phenotype path and the gene path (achieved by multiplying *P* on the left and *G* on the right in each step), the algorithm explores the CBGs weighted by a decay factor *α* ∈ (0,1). The decay factor down-weights the importance of a CBG as the path length is getting longer. Here, the matrix multiplications (*P*_(*m*×*m*)_⋅*A*_(*m*×*n*)_⋅*G*_(*n*×*n*)_) mimic jumps on the phenotype network, the gene network and the association network. In the first step, each element *PAG*_(*i*, *j*)_ represents the number of CBGs obtained by connecting a target phenotype *i* to a candidate gene *j* with phenotype or gene paths length 1. If we ignore the decay factor for now, more generally, after *t* steps of multiplication *P*…(*P*(*P*⋅*A*⋅*G*)*G*)…*G* = *P* ^*t*^ *AG* ^*t*^, CBG patterns with path length *t* can be evaluated. To achieve the best solution *R*_(*m*×*n*)_, we formulated the problem as *R* = *PRG*, assuming *P* is column-normalized, *G* is row-normalized, and the elements in *R* add to 1. *PRG* can be rewritten in a vector form $P⊗G\hat{R}$ where ⊗ is Kronecker product and $\hat{R}$ is the vector obtained by concatenating the columns in *R*. Each step of bi-random walk is the same as a random walk on the Markov matrix *P*⊗*G*. Thus, the step of evaluating the CBGs is identical to using power method to find the stationary distribution of *P*⊗*G*.

### Bi-Random Walk Algorithm

However, since only the CBGs of small path lengths are informative for predicting associations, excessively counting CBGs of long path lengths could introduce false positives. [23] suggested that genes within two-steps in a PPI network are more functional cohesion. Moreover, the phenotype similarity network and the gene network contain different topologies and structures, and thus, the optimal number of random walk steps might be different on the two networks. To address the problem, we restrict the number of random walk steps on the two sides by introducing two parameters (*l*, *r*) as the numbers of maximal iterations in the following left/right random walk on the networks,

$$\begin{array}{ccc} \text{Left}\;\text{Walk}:\;R_{t} & = & \alpha P\cdot R_{t-1}+\left(1-\alpha \right)A\\ \text{Right}\;\text{Walk}:\;R_{t} & = & \alpha R_{t-1}\cdot G+\left(1-\alpha \right)A \end{array}$$ (1)

The BiRW algorithm takes *P*, *G*, *A*, the decay factor *α*, left/right walk steps *l* and *r* as the inputs and outputs the predicted associations *R*. The BiRW algorithm is outlined as follows,

BiRW(*P*, *G*, *A*, *α*, *l*, *r*)

 1 $\bar{P}=D_{P}^{-\frac{1}{2}}PD_{P}^{-\frac{1}{2}}$

 2 $\bar{G}=D_{G}^{-\frac{1}{2}}GD_{G}^{-\frac{1}{2}}$

 3 $R_{0}=A=\frac{A}{sum(A)}$

 4 **for** *t* = 1 **to** *max*(*l*, *r*)

 5  **if** *t* < = *l*

 6   $\;R_{t\_left}=\alpha \bar{P}\cdot R_{t-1}+(1-\alpha )A$

 7  **if** *t* < = *r*

 8   $R_{t\_right}=\alpha R_{t-1}\cdot \bar{G}+(1-\alpha )A$

 9  *R*_*t*_ = (*δ*_*t* ≤ *l*_⋅*R*_*t*_*left*_+*δ*_*t* ≤ *r*_⋅*R*_*t*_*right*_)/(*δ*_*t* ≤ *l*_+*δ*_*t* ≤ *r*_)

 10 **return** (*R*)

Note that *P* is normalized as $\bar{P}=D_{P}^{-\frac{1}{2}}PD_{P}^{-\frac{1}{2}}$ where *D*_*P*_ is a diagonal matrix with diagonal elements *D*_*Pii*_ = ∑_*j*_ *P*_*ij*_, and $\bar{G}$ is the same normalized from *G*. *A* is normalized with elements adding up to 1. Line 6 and line 8 are the left and right random walks. In Line 9, the propagation results are combined, where *δ*_*t* ≤ *x*_ is 1 if *t* ≤ *x* and 0 otherwise. The algorithm will terminate as the number of iterations exceeds *max*(*l*, *r*).

### Regularization Framework of Bi-Random Walk

Clearly, BiRW explores CBG patterns on the phenotype network and the gene network. The algorithm assumes that the probability of a phenotype being associated with a gene is proportional to the the number of weighted CBGs that are explored by bi-random walk and connecting the phenotype and the gene. An equivalent statement is that a potential association between a phenotype and a gene is evaluated by its distance to the other associations in the phenotype network and the gene network. Actually, the closer the potential association to the other associations, the more highly weighted CBGs will be created by this association. Thus, BiRW is a global strategy to complete the association map, instead of prioritizing genes for a specific disease phenotype. Accordingly, it provides a global optimality in the predicted associations. The mathematical interpretation of BiRW is by connecting a phenotype and a gene how many weighted new CBG patterns are curated [22].

Given phenotype network *P* and gene network *G*, *P*⊗*G* is the Kronecker product of *P* and *G*. Each *P*⊗*G*_(*i*, *u*), (*j*, *v*)_ is 1 if *P*_*i*, *j*_ = 1 and *G*_*u*, *v*_ = 1, in other words phenotype *i* and *j* are neighbors and gene *u* and *v* are also neighbors, and otherwise 0. BiRW learns an association matrix *R* to minimize the following regularization framework,

$$\begin{array}{c} \alpha \sum_{u,v,i,j}{\left(P⊗G\right)}_{\left(i,u),(j,v\right)}{\left(R_{i,u}-R_{j,v}\right)}^{2}+\left(1-\alpha \right)\sum_{i,u}{\left(R_{i,u}-A_{i,u}\right)}^{2}. \end{array}$$

In this regularization framework, the first term enforce a smoothness on *R* where phenotypes (*i*, *j*) and gene (*u*, *v*) should form a CBG with phenotype *i* aligned with phenotype *j* and gene *u* aligned with gene *v* when (*i*, *j*) are neighbors and (*u*, *v*) are also neighbors. The second term uses prior knowledge *A* as a regularization term. The matrix form of the above objective function is

$$\begin{array}{c} \max_{R}\;\alpha {\hat{R}}^{T}\left(D-\left(P⊗G\right)\right)\hat{R}+\left(1-\alpha \right){\left|\right|\hat{R}-\hat{A}\left|\right|}^{2}, \end{array}$$ (2)

where *D* is the diagonal matrix with the row sum of *P*⊗*G* as the diagonal entries. This objective function is minimized by BiRW [22][24]. IsoRank algorithm is an bi-random walk algorithm for global network alignment between PPI networks [25, 26]. While balanced BiRW is identical to IsoRank, BiRW explicitly interprets iterative random walks on the two networks as steps to extend the CBG paths, and thus, introduces the flexibility to perform unbalanced left/right walk to capture CBG patterns more precisely.

### Related Work

BiRW is related to several other network-based algorithms for disease gene prioritization [9, 11–18]. CIPHER scores the association between a gene *g* and the phenotype *p* by computing the correlation coefficient between the gene-phenotype profile of *g* and the phenotype similarity profile of *p* [13]. The gene-phenotype profile of *g* is computed by a logistic function based on the direct neighbors of *g* or the path length between *g* and causal genes of each phenotype. PRINCE performs label propagation on the PPI network to prioritize disease genes [15]. The initial probabilities on the gene nodes are normalized from the causative genes of the nearest neighbors of the query phenotype *p* chosen by a logistic function. The initial scores are propagated in the stochastic matrix normalized from the PPI network. After convergence, the unique solution of label propagation is used to rank the genes. RWRH [16] runs the same label propagation algorithm on the combined heterogeneous network of all the three networks to rank genes for a query phenotype. MINProp [14] is based on a principled way to integrate three networks in an optimization framework and performs iterative label propagation on each individual subnetwork. MAXIF [18] maximizes the information flow in the phenome-genome association network to identify the sink genes for a source phenotype.

These disease gene prioritization algorithms rank genes based on their predicted association against a particular query phenotype *p* while BiRW is a global approach which identifies the missing associations of all the phenotypes simultaneously. Conceptually, BiRW is a multi-task learning method for phenome-genome prediction while the previous methods are single-task learning methods for phenotype-wise association prediction, which do not explore the relation between the predicted associations across the phenotypes. The mathematical difference between BiRW and the other algorithms lies in the formulation of using the known associations in *A*. CIPHER and PRINCE use the known associations to decide an initial set of genes that are associated with a query phenotype. RWRH, MINProp and MAXIF directly use *A* as part of the large network for random walk or maximum flow computation. BiRW treats the associations *R* as the target variable and the known association *A* as a regularization of *R*, intuitively, because *A* is only partially known and most of the zero entires of *A* are “unknown” instead of “no association”. Thus, using *A* as a regularization instead of directly as part of the network for graph structure-based learning is probably a more rigorous modeling because the incompleteness of the bipartite network might mislead the random walk.

## Results

In the experiments, we first analyzed OMIM disease gene associations and MGI mouse phenotype-gene associations by reporting the statistics of CBG patterns in the networks. We then performed cross-validation and test of an independent test set of OMIM associations to evaluate the performance of BiRW. BiRW was also validated by cross-validation on the MGI mouse phenotype-gene associations to test the applicability to mouse phenome-genome analysis. Statistical analysis was performed on randomized OMIM data to validate the statistical significance of the results. Finally, we analyzed the predicted OMIM disease phenome-genome association by examining association modules by each disease class.

### Data Preparation

The OMIM disease phenotype network is an undirected graph with 5080 vertices representing OMIM disease phenotypes, and edges weighted in [0, 1]. The edge weights measure the similarity between two phenotypes by their overlap in the text and the clinical synopsis in OMIM records, calculated by text mining [6]. The disease-gene associations are represented by an undirected bipartite graph with edges connecting phenotype nodes with their causative gene nodes. Two versions (May-2007 Version and July-2014 Version) of OMIM associations were used in the experiments. May-2007 Version contains 1393 associations between 1126 disease phenotypes and 916 genes, and July-2014 Version contains 1987 associations between 1512 disease phenotypes and 1265 genes. Human protein-protein interaction (PPI) network was obtained from HPRD [7]. The PPI network contains 34,364 curated binary interactions between 8919 genes.

The mouse phenotype similarity network was constructed by Mammalian Phenotype (MP) ontology terms downloaded from MGI [5]. As suggested by [27] that the most frequent annotations are at level 5 in MP ontology, 2612 ontology terms at level 5 were selected from the 10271 MP ontologies in the OBO file. The similarity is calculated for each pair of phenotype ontologies by Jaccard similarity coefficient. The mouse phenotype-gene associations were extracted from file “MGI_Geno_Disease.rpt” downloaded from MGI website, in which 9798 phenotype-gene associations between 1261 phenotypes at level 5 and 815 genes were kept. Note that to utilize the ontology structure, a level-5 phenotype is considered to be associated with a gene if the phenotype node itself or any decedent node of the node is associated with the gene. The mouse PPI network downloaded from BioGrid [28] (version 3.2.113) was used as the gene network, which contains 19,402 PPIs between 8006 genes.

### Analysis of OMIM and MGI Associations by CBG Statistics

To analyze the CBGs in the OMIM disease phenome-genome association network, we calculated the frequencies of the CBG patterns in OMIM (July-2014) phenotype-gene association network. In the phenotype similarity network, the five nearest nodes of each node were selected as neighbors [16]. We first report the percentage of the 1987 OMIM associations covered in CBGs categorized by the path length in Fig 2. Only 34.68% of the associations are covered by CBG patterns of path length 1 (Fig 2A). When larger CBGs are considered, the percentage is significantly higher. Specifically, 62% of the associations are covered by CBG patterns of path length up to 2 and 87.72% of the associations are covered by CBG of path length up to 3. When we randomly created 1987 links between 5080 disease phenotypes and 8919 genes, there is 4.1 average occurrences of CBG of path length 1 in 1000 runs (S1 Table). The significance is expected because the diameter of the phenotype network is 33, and there are many components in the PPI network with the largest diameter 14.

To further analyze the relation between CBGs and disease phenotypes, we calculated the CBG statistics for the 21 disease phenotype classes that are manually curated by [29]. The statistics are reported in Table 1. Some of the classes such as bone diseases and cancers have a high CBG coverage on their OMIM associations while some other classes such as metabolic diseases, ear-nose-throat diseases and psychiatric diseases have lower coverage. For example, above 98% associations in cancers, nutritional diseases and bone diseases, are covered by CBGs with path length up to 3, while only 63.41% of those in metabolic disease are covered. A more detailed break-down of the CBGs in each disease class by lengths is also given in a table in S2 Fig.

**Table 1:** CBGs in OMIM phenotype-gene associations. OMIM associations in 21 disease classes that are covered by CBGs up to a certain length (1–3). The coverage by percentage and the AUC in cross-validation are reported.

Disease Classes	CBG Len = 1	CBG Len ≤ 2	CBG Len ≤ 3	Assoc #	Coverage CBG1 ∼ 2	Coverage CBG1 ∼ 3	Pheno #	AUC_1000_
Bone	21	33	40	40	82.50%	100.00%	31	0.8236
Cancer	93	192	215	219	87.67%	98.17%	67	0.8218
Cardiovascular	25	57	86	88	64.77%	97.73%	58	0.6475
Connectivetissue	11	25	31	33	75.76%	93.94%	24	0.6731
Dermatological	50	61	77	83	73.49%	92.77%	66	0.7565
Developmental	15	24	33	36	66.67%	91.67%	26	0.6442
Ear, Nose, Throat	6	8	19	23	34.78%	82.61%	20	0.5771
Endocrine	33	49	72	79	62.03%	91.14%	47	0.6276
Gastrointestinal	10	16	18	23	69.57%	78.26%	16	0.6692
Hematological	31	48	60	66	72.73%	90.91%	55	0.6388
Immunological	24	52	68	72	72.22%	94.44%	41	0.4695
Metabolic	35	46	78	123	37.40%	63.41%	114	0.2948
Muscular	43	56	64	68	82.35%	94.12%	54	0.7451
Neurological	61	104	162	186	55.91%	87.10%	150	0.5216
Nutritional	8	13	17	17	76.47%	100.00%	3	0.5381
Ophthamological	25	42	51	71	59.15%	71.83%	68	0.6356
Psychiatric	0	8	18	25	32.00%	72.00%	10	0.3610
Renal	16	25	33	38	65.79%	86.84%	33	0.5606
Respiratory	10	18	25	28	64.29%	89.29%	8	0.3523
Skeletal	28	45	53	59	76.27%	89.83%	54	0.8236
Multiple	48	83	117	124	66.94%	94.35%	108	0.6256

The frequencies of the CBG patterns were also calculated in the MGI mouse phenome-genome association network. When ten nearest nodes of each phenotype node were selected as neighbors, 25.48%, 51.42% and 65.67% of the associations are covered by CBGs of length up to 1, 2 and 3 respectively (Fig 2A), with respect to an average of 3.17%, 10.22% and 20.41% in the networks with randomized phenotype-gene associations. Although lower than the OMIM associations, the statistics still suggest significant enrichment of CBGs (S11 Table).

### Comparison with Other Methods by Phenotype-gene Association Prediction

BiRW was compared to CIPHER [13], PRINCE [15] and RWRH [16] in a 100-fold cross-validation on the OMIM May-2007 Version, and prediction of the associations in an independent set of associations added into OMIM between May-2007 and July-2014. The three algorithms were applied to predict the disease genes for each phenotype and the predictions are compared with the results of BiRW phenotype-wise. The 1126 disease phenotypes with at least one known causal gene in OMIM version May-2007 were randomly divided into 100 folds. In each cross-validation trial, the OMIM associations of the 1% disease phenotypes in a subset were removed, and then used as queries to rank the candidate genes.

AUCs (Area Under the Curve of Receiver Operating Characteristic) was used as the global performance measure. The higher the target genes of a query phenotype in the ranking, the better the performance. We reported the AUC with up to 50, 100, 300, 500 and 1000 false positives since the top part of AUC is more important. In addition, we also report the recall of the test phenotypes, which measures how many target genes are ranked within top *k*. For more detail of the experiment setup, see supplementary document and [22].

**Fig 4:**
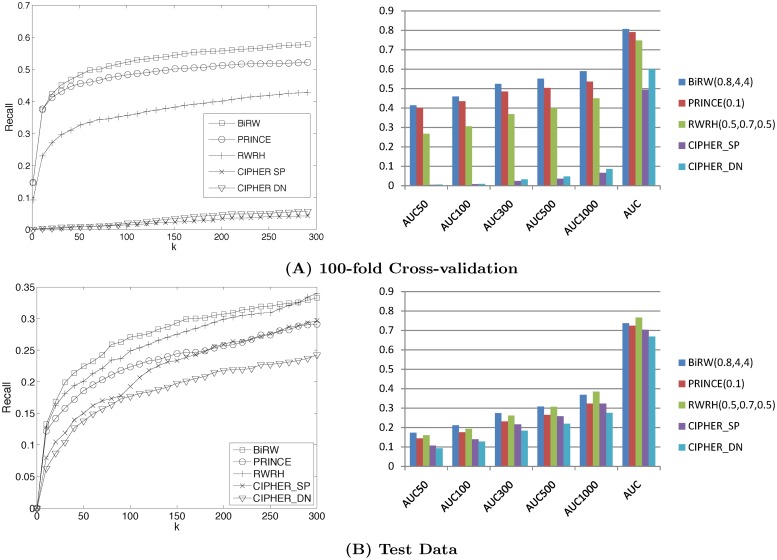
Performance of predicting OMIM associations. The plots compare the performance of all the methods on predicting the target genes of query phenotypes in cross-validation (A) and testing on the test set (B). The left plots show the recalls at different top-*k* cutoff. The right plots compares the average AUCs across all the test phenotypes by the compared methods.

The results produced by the best parameters in the cross-validation of each method is reported in Fig 4A (*l* = 4, *r* = 4 and *α* = 0.8 for BiRW; *α* = 0.1 for PRINCE; (0.5,0.7,0.5) for RWRH). The detailed parameter tuning for BiRW, PRINCE and CIPHER are given in S2–S4 Tables. The recall are reported in the left plots in Fig 4 and the complete results measured by AUCs up to different false positives are reported in the right plots in Fig 4 and S5 Table. We also measured the statistical significance of the difference in AUC_50_ and AUC_100_ by paired *t*-test. The *p*-values are reported in S7 Table. Clearly, BiRW performs significantly better than all the other methods at the significance level 0.05. PRINCE also gave decent prediction performance although BiRW consistently outperformed PRINCE in all the measures. RWRH, CIPHER DN (direct neighbor) and SP (shortest path) produced inferior results in this experiment. The possible reason for the worse results of CIPHER might be because the associations of the test phenotypes were all removed (called *ab initio* experiment) and each cross-validation held out a significant number of known associations. Thus, no direct neighbors are available for the correlation calculation for many phenotype queries by CHPHER. PRINCE, RWRH and BiRW worked much better than CIPHER SP and CIPHER DN because label propagation and bi-random walk both explore more global information of the networks. In the last column of Table 1, the average AUC of the phenotypes in each disease class by BiRW are also reported. As expected, there is a strong correlation (Pearson’s correlation = 0.798 between AUC and CBG2 coverage) between the CBG coverage and the AUCs. For example, the cancer and bone classes have both high coverage and AUC, while psychiatric and metabolic classes have both low coverage and AUC.

With the best parameter from cross-validation, the methods were used to predict the new associations of 656 phenotypes in OMIM July-2014 Version. The results are reported in Fig 4B. BiRW consistently outperformed the other methods although RWRH performed better on the holdout set than the cross-validation. Interestingly, PRINCE performed worse in the test than the cross-validation, which might suggest a possible bias when the neighbors’ disease genes are directly initialized as true disease genes. CIPHER DN and SP performed better on the test set since the test cases have other known disease genes and thus, are not as difficult as those in cross-validation. A more detailed comparison of BiRW, RWRH, PRINCE and CIPHER by AUCs is given in S6 Table. The *p*-values by paired *t*-test are reported in S8 Table. Clearly, BiRW and RWRH performed significantly better than all other methods while BiRW performs better at the the starting part of the AUCs.

**Fig 5:**
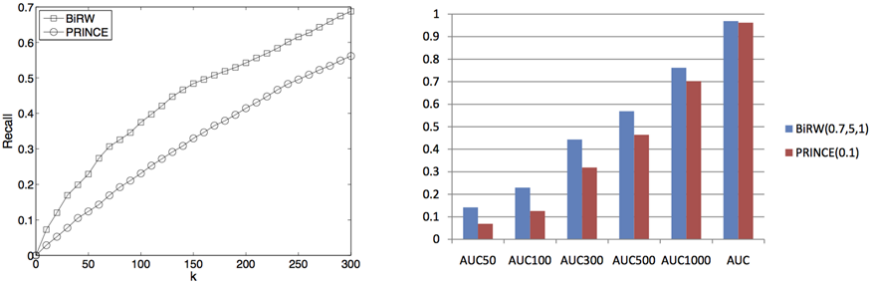
Performance of predicting mouse phenotype-gene associations. The plots compare the performance of BiRW and PRINCE on predicting the target genes of query mouse phenotypes in 100-fold cross-validation. The left plot shows the recalls at different top-*k* cutoff. The right plot compares the average AUCs across all the test phenotypes by the compared methods.

The same 100-fold cross-validation was performed to test BiRW and PRINCE with mouse phenotype-gene network. The best parameters are *α* = 0.7, *l* = 5, *r* = 1 for BiRW and *α* = 0.1 for PRINCE based on a grid search (S9 and S10 Tables). The performance of predicting the mouse phenotype-gene associations are shown in Fig 5. BiRW clearly outperforms PRINCE in all measures. The prediction performance in mouse data is comparable to or better than the results in human data when larger number of false positives are allowed. The AUC_50_ and AUC_100_ are much lower in the mouse data which indicates that the lower CBG coverage in the MGI phenome-genome association network plays a role (Fig 2). In the last column of Table 2, the average AUC of the phenotypes in each branch at MPO level one by BiRW are also reported. The Pearson’s correlation = 0.38 between the CBG coverage and AUC, which is lower than the human data.

**Table 2:** CBGs in mouse phenotype-gene association network. Phenotype-gene associations grouped by 26 MPO terms at level 1 that are covered by CBGs up to a certain length. The coverage by percentage and the AUCs in cross-validation are reported.

MPO Terms in Level 1	CBG Len = 1	CBG Len ≤ 2	CBG Len ≤ 3	Assoc #	Coverage CBG1 ∼ 2	Coverage CBG1 ∼ 3	Pheno #	AUC_1000_
pigmentation pheno.	8	14	19	35	40.00%	54.29%	4	0.7350
tumorigenesis	44	104	129	188	55.32%	68.62%	5	0.6802
nervous sys. pheno.	180	407	562	940	43.30%	59.79%	141	0.7478
renal/urinary sys. pheno.	85	188	266	410	45.85%	64.88%	89	0.7474
muscle pheno.	99	127	147	199	63.82%	73.87%	45	0.7623
liver/biliary sys. pheno.	9	42	71	98	42.86%	72.45%	15	0.7189
limbs/digits/tail pheno.	94	121	141	185	65.41%	76.22%	22	0.8074
adipose tissue pheno.	19	44	51	71	61.97%	71.83%	22	0.7547
homeostasis/metabolism pheno.	174	418	572	858	48.72%	66.67%	127	0.7707
hearing/vestibular/ear pheno.	78	124	161	259	47.88%	62.16%	40	0.8053
growth/size/body pheno.	55	150	186	332	45.18%	56.02%	8	0.6964
endocrine/exocrine gland pheno.	61	169	228	324	52.16%	70.37%	58	0.7518
embryogenesis pheno.	28	58	82	128	45.31%	64.06%	49	0.7644
digestive/alimentary pheno.	47	97	131	195	49.74%	67.18%	47	0.7247
craniofacial pheno.	65	153	220	350	43.71%	62.86%	25	0.7261
cellular pheno.	36	88	119	209	42.11%	56.94%	48	0.7618
cardiovascular sys. pheno.	210	414	520	788	52.54%	65.99%	115	0.7709
behavior/neurological pheno.	222	436	534	873	49.94%	61.17%	71	0.7777
immune sys. pheno.	377	656	784	1092	60.07%	71.79%	83	0.7747
respiratory sys. pheno.	21	56	83	150	37.33%	55.33%	37	0.7447
reproductive sys. pheno.	136	229	263	322	71.12%	81.68%	36	0.7741
skeleton pheno.	175	357	459	664	53.77%	69.13%	76	0.8005
vision/eye pheno.	103	159	190	286	55.59%	66.43%	44	0.7790
hematopoietic sys. pheno.	39	143	184	298	47.99%	61.74%	16	0.7596
mortality/aging	107	232	267	432	53.70%	61.81%	10	0.7269
integument pheno.	25	52	65	112	46.43%	58.04%	28	0.7819

### Robustness Analysis with Unbiased PPI Network

**Fig 6:**
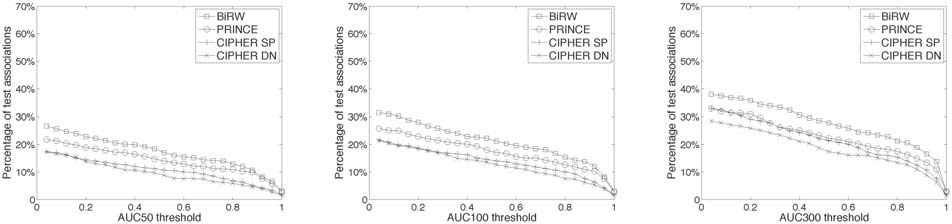
Performance of predicting OMIM associations with the unbiased PPI network in OMIM July-2014.

It is known that well-studied disease proteins tend to have more interactions in the PPI network and this degree bias could potentially lead to the good performance of the network-based methods. To test whether the methods are robust to the degree bias, we repeated the experiments on an extended PPI network. The extended PPI network with the same degree of interactions for each protein was generated to assess the influence of the degree bias. The extended PPI network was combined from HPRD, OPHID, BIND, and MINT database contain 72,431 undirected binary interactions between 14,433 human proteins [13]. The results are reported in Fig 6 and Table 3. The BiRW algorithm consistently outperformed the baselines. With the replacement by the unbiased PPI network, all the methods performed actually similarly as in the original experiment. This experiment on the extended PPI network suggests that BiRW is also robust to the degree bias in the networks.

**Table 3:** AUC scores of predicting new disease genes in OMIM July-2014 with the unbiased PPI network.

	AUC_50_	AUC_100_	AUC_300_	AUC_500_	AUC_1000_	AUC
BiRW(0.8, 4, 4)	0.1736	0.2127	0.2787	0.3135	0.3729	0.7375
PRINCE(0.1)	0.1444	0.1758	0.2297	0.2615	0.3145	0.7212
CIPHER SP	0.1072	0.1404	0.2168	0.2583	0.3230	0.7034
CIPHER DN	0.0958	0.1290	0.1880	0.2214	0.2791	0.6721

### Analysis of Gastrointestinal Disease-gene Modules

Identification of the essential biological processes of diseases can lead to new insights into possible pathogenic mechanisms, and development of efficient targeted therapeutics. However, the current known associations between human disease traits and genes are too sparse to support such studies. BiRW identifies the essential associations for similar disease phenotypes and tend to generate bi-modules for a collection of phenotypes in the same disease class. Such bi-modules could help discover the core biological mechanisms underlying the human disease classes. To derive such bi-module for a disease class, we collected the known disease genes and the top 3% high-association genes (e.g. top 74 genes) of each phenotype in the predicted phenome-genome association, from which those genes that occurred as a disease gene of at least five phenotypes in the disease class (or all the phenotypes if the disease class contains less than five phenotypes) were included in the module. The genes associated with only a few phenotypes in the disease class were filtered to keep the modules dense. Each disease-gene association module describes the associations between the phenotypes in a disease class and the predicted frequent disease genes of the phenotypes. We focused on the analysis of the gastrointestinal disease-gene module as an example. Note that this analysis was only performed by all the associations available in May, 2010.

The known disease phenotype-gene associations in the gastrointestinal disease class are very sparse; therefore, no enriched GO biological processes (corrected *p*-value < 0.05) were found with standard gene set enrichment analysis. Indeed, most gastrointestinal disease phenotypes do not share any disease causative genes in common in OMIM. This observation agrees with the findings from recent studies in GWAS [30, 31], which reported that phenotypically similar diseases that are gastrointestinal-related do not tend to share their disease genes. These previous studies also hypothesized that, due to the unique topological characteristics of the gastrointestinal disease susceptibility genes, the existing network-based methods would also fail to reveal any common disease genes for understanding the underlying biological mechanisms of gastrointestinal diseases.

**Fig 7:**
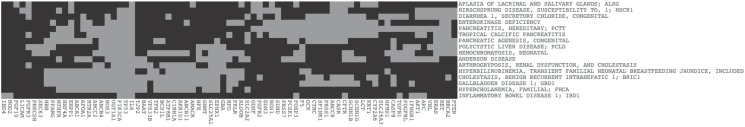
Gastrointestinal disease phenotype-gene association module. The predicted gastrointestinal disease module of associations (grey color) between 64 disease genes including the 22 known disease genes associated with 16 gastrointestinal disease phenotypes.

On contrary, BiRW identified a gastrointestinal disease-gene module that shows many common genes across the phenotypes in the disease class in Fig 7. Some interesting examples are IL6, TP53, and PIK3CA. IL6 is a previously known causative gene of inflammatory bowel disease but not other gastrointestinal disease phenotypes. However, recent studies discovered that IL6 is involved with other gastrointestinal related disease phenotypes including pancreatitis [32], polycystic liver disease [33], salivary glands [34], congenital diarrhea [35], pancreatic agenesis [36] and gallbladder disease [37]. TP53 and PIK3CA are also not known for association with any gastrointestinal-related disease phenotype but it was recently determined that TP53 and PIK3CA play a role in developing gallbladder [38], pancreatic agenesis [39] and inflammatory bowel disease [40, 41].

We further studied the functional roles of the predicted disease genes in each module with enrichment analysis against Gene Ontology biological processes [42] using DAVID [43]. The enrichment *p*-values were adjusted by *Bonferroni* correction for multiple testing. The GO biological processes enriched by the predicted disease genes in each module with *p*-value ≤ 0.05 are reported in S3 Fig. Across 19 human disease classes. Nutritional, and Ear, Nose, Throat disease classes were left out since there was no predicted frequent disease genes that passed the selection criteria. Many GO biological process terms known for relevance with the disease classes are significantly enriched, such as “cell migration”, “cell proliferation”, and “homeostatic process” for gastrointestinal diseases, and “cell proliferation”, “programmed cell death”, and “apoptosis” for cancers, while these biological process terms cannot be readily identified by very sparse existing disease-gene associations. Another notable example is the enrichment of “behavior”, “synaptic transmission”, and “transmission of nerve impulse” by the causative genes of psychiatric diseases. Recent studies showed that regulation of synaptic transmission and transmission of nerve impulse are associated with psychiatric disease phenotypes such as autism [44].

## Discussion

Description of the full collection of phenotypes (called phenome) such as PhenomicDB, PhenoGO and Gramene Ontologies are now becoming more stabilized and systematic. The next step is to develop computational techniques for a global inference phenome-genome association based on the previous discoveries and the phenome information. Towards this goal, we analyzed the patterns of OMIM and MGI phenotype-gene associations by correlating the associations with phenotype similarity network and gene network. We showed that majority of the known associations are part of short circular bigraphs. This non-random pattern provides the foundation for deriving new associations based on the linkages in the networks. The BiRW algorithm is specifically designed to capture the circular bigraphs, and thus, can more reliably reconstruct the complete phenome-genome association. Functional analysis of the reconstructed phenome-genome association by disease classes revealed a global map between GO biological processes and human disease classes. In future, we plan to further investigate the relation between disease classes and the GO biological processes to understand the common molecular mechanisms of human diseases.

The experiments in 100-fold cross-validation are designed to mimic the environment of phenome-genome prediction, where associations with genes are missing for multiple phenotypes. We demonstrated that BiRW will handle the phenome-genome prediction better than algorithms that prioritize genes for each individual phenotype. The results in the study on mouse data suggest that network-based phenotype-gene association analysis is generally applicable to other species. The major challenge lies in the selection and preparation of phenotype and phenotype-gene association data. For example, most of the phenotypes are organized in an ontology and representing phenotype relations as a network for random walk is not optimal. In future, we plan to design better algorithms to integrate ontologies in the same analyses.

A challenge of applying BiRW is the choice of the random walk steps in the two coupled networks. Prior knowledge is always helpful in determining the effective steps. For example, in the PPI network less than five steps are enough for gene function prediction. In general, the optimal steps depends on the density of the networks and the density of the node cluster neighborhoods. In the future, we also plan to investigate the general strategies for choosing the number of steps.

The major limitation of network-based phenotype-gene association methods including BiRW is the strong dependence on the network connections. For sparse networks with many isolated small components, random walk or network propagation are less useful since no global information can be introduced into the small components. As such, when a novel association is between a phenotype and a gene in a small component with no known phenotype associations, no prediction can be made. To overcome the limitation, one possibility is to integrate other genomic data such as gene expressions or SNPs in a patient cohort or a population as features to classify the genes. In the future, we plan to develop new methods that can incorporate these additional genomic data.

Another potential limitation of BiRW is the weak assumption on clusters. BiRW relies on small graph patterns which are not necessarily equivalent to clusters in the phenome-genome network. The interpretation of the bi-random walk result is not as straight-forward as those based on propagations on node values. For example, previous studies [10, 19] using protein complexes to group proteins can naturally identify protein complexes associated with a disease phenotype as disease gene modules. A possible extension of BiRW is to impose a complex-phenotype relation as constraints on the bi-random walk to introduce local clusters for better interpretation.

## Supporting Information

S1 Fig Examples of circular bigraphs in OMIM disease phenotype-gene association network. Each circular bigraph is named by ‘CBG *l*:*r*’, where *l* is the length of the phenotype path and *r* is the length of the gene path.
(TIFF)

S2 Fig Circular bigraphs in 21 disease categories. The associations in each disease class are categorized by the length of the CBGs covering the association.
(TIFF)

S3 Fig The genetic landscape of human diseases. Association between GO biological processes and 19 human disease classes. The gray entries incidence a biological process and a disease class if the biological process is significantly enriched by the causative genes of the disease class.
(TIFF)

S1 Table CBG statistics on human phenome-genome network (July 2014). CBG analysis with randomized phenotype-gene network are reported for comparison.
(PDF)

S2 Table Parameter tuning for BiRW with 100-fold cross-validation on OMIM May-2007. (A) This table reports the average AUCs across all the phenotypes by converged balanced BiRW(*l* = *r*) with different parameter *α*. The number of phenotypes with AUC more than 0.9 and 0.7 are also reported. The chosen *α* = 0.8 is among the best *α*s.
(B) The tables report the AUC_50_, AUC_100_ and AUC_300_ by BiRW with the step parameter *l* and *r* (range from 1 to 5), where the parameter *α* is fixed as 0.8 based on the result in Fig 1A. The bold AUC scores are the best ones. Based on the results, the BiRW parameters are chosen as *α* = 0.8, *l* = 4 and *r* = 4 in 100-fold cross-validation.
(PDF)

S3 Table Parameter tuning for PRINCE in 100-fold cross-validation on OMIM May-2007. This table reports the average AUCs across all phenotypes for PRINCE with the balance parameter *α*.
(PDF)

S4 Table Performance of CIPHER by 100-fold cross-validation on OMIM May-2007. The table reports the average AUCs across all phenotypes for CIPHER SP (shortest path in PPI) and CIPHER DN (direct neighbor).
(PDF)

S5 Table AUCs of the cross-validation on OMIM May-2007. The table reports a comparison of the ranking results by BiRW and four other methods, PRINCE, RWRH, CIPHER SP and CIPHER DN. The parameters *α*, *l* and *r* of BiRW are set by the experimental results in 100-fold cross-validation. AUCs up to 50, 100, 300, 500, 1000 and all false positives are reported.
(PDF)

S6 Table AUCs of the prediction of the new disease genes in OMIM July-2014. The table reports a comparison of the ranking results by BiRW and 4 baselines, PRINCE, RWRH, CIPHER SP and CIPHER DN. The parameters *α*, *l* and *r* of BiRW are set by the experimental results in 100-fold cross-validation. AUCs up to 50, 100, 300, 500, 1000 and all false positives are reported.
(PDF)

S7 Table A pairwise comparison by paired *t*-test of the ranking results in 100-fold cross-validation based on AUCs. (PDF)

S8 Table A pairwise comparison by paired *t*-test of the ranking results in test based on AUCs. (PDF)

S9 Table Performance of BiRW with 100-fold cross-validation on mouse phenome-genome network. The parameters *α*, *l* and *r* of BiRW are tuned, and the best AUCs(up to 50, 100, 300, 500, 1000 and all false positives) with parameter *α* are reported. Based on the AUC_50_ results, the BiRW parameters for mouse are chosen as *α* = 0.7, *l* = 5 and *r* = 1 in 100-fold cross-validation
(PDF)

S10 Table AUCs of PRINCE in 100-fold cross-validation on mouse phenome-genome network. Parameter *α* ranges from 0.01 to 0.9 for tuning better. AUCs up to 50, 100, 300, 500, 1000 and all false positives are reported.
(PDF)

S11 Table CBG statistics on mouse phenome-genome network. CBG analysis with randomized phenotype-gene network are reported for comparison.
(PDF)
