# Supplementary figures and images for: Network-based Phenome-Genome Association Prediction by Bi-Random Walk
Source: PLoS One. 2015 May 1;10(5):e0125138. doi: 10.1371/journal.pone.0125138 (PMC4416812; doi:10.1371/journal.pone.0125138)

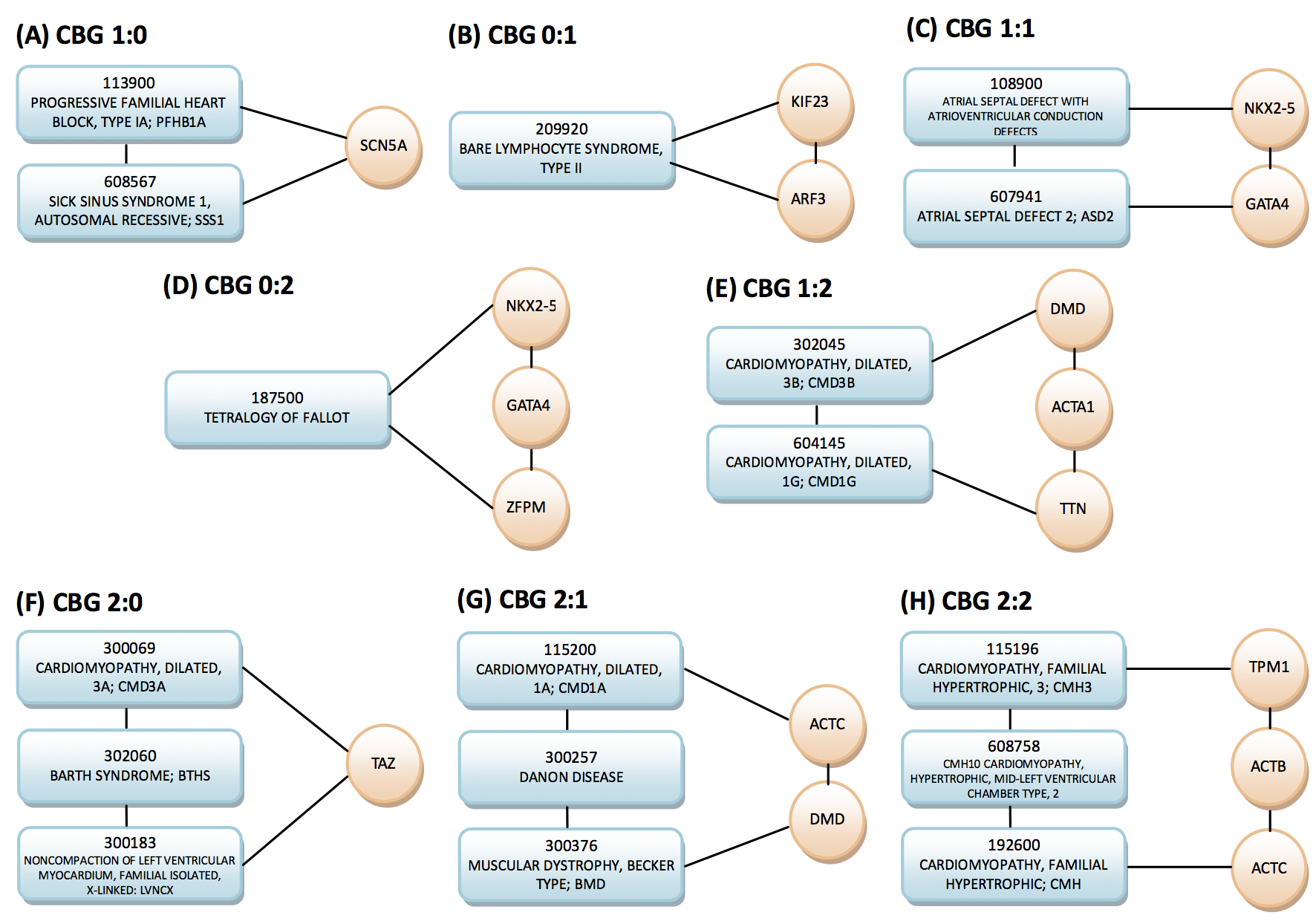

Supplement: S1 Fig — Each circular bigraph is named by ‘CBG l:r’, where l is the length of the phenotype path and r is the length of the gene path. (TIFF) [file pone.0125138.s001.tiff]

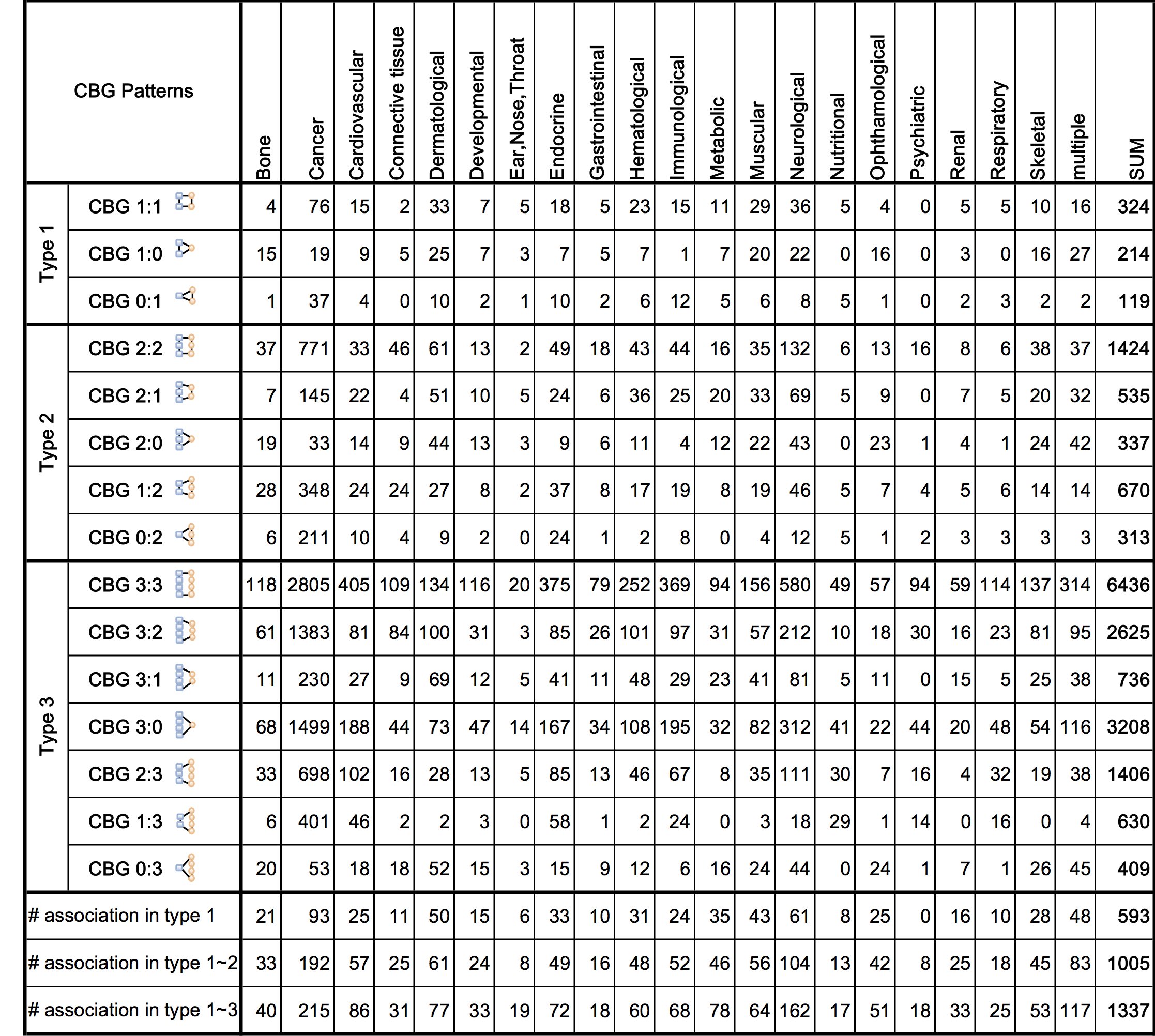

Supplement: S2 Fig — The associations in each disease class are categorized by the length of the CBGs covering the association. (TIFF) [file pone.0125138.s002.tiff]

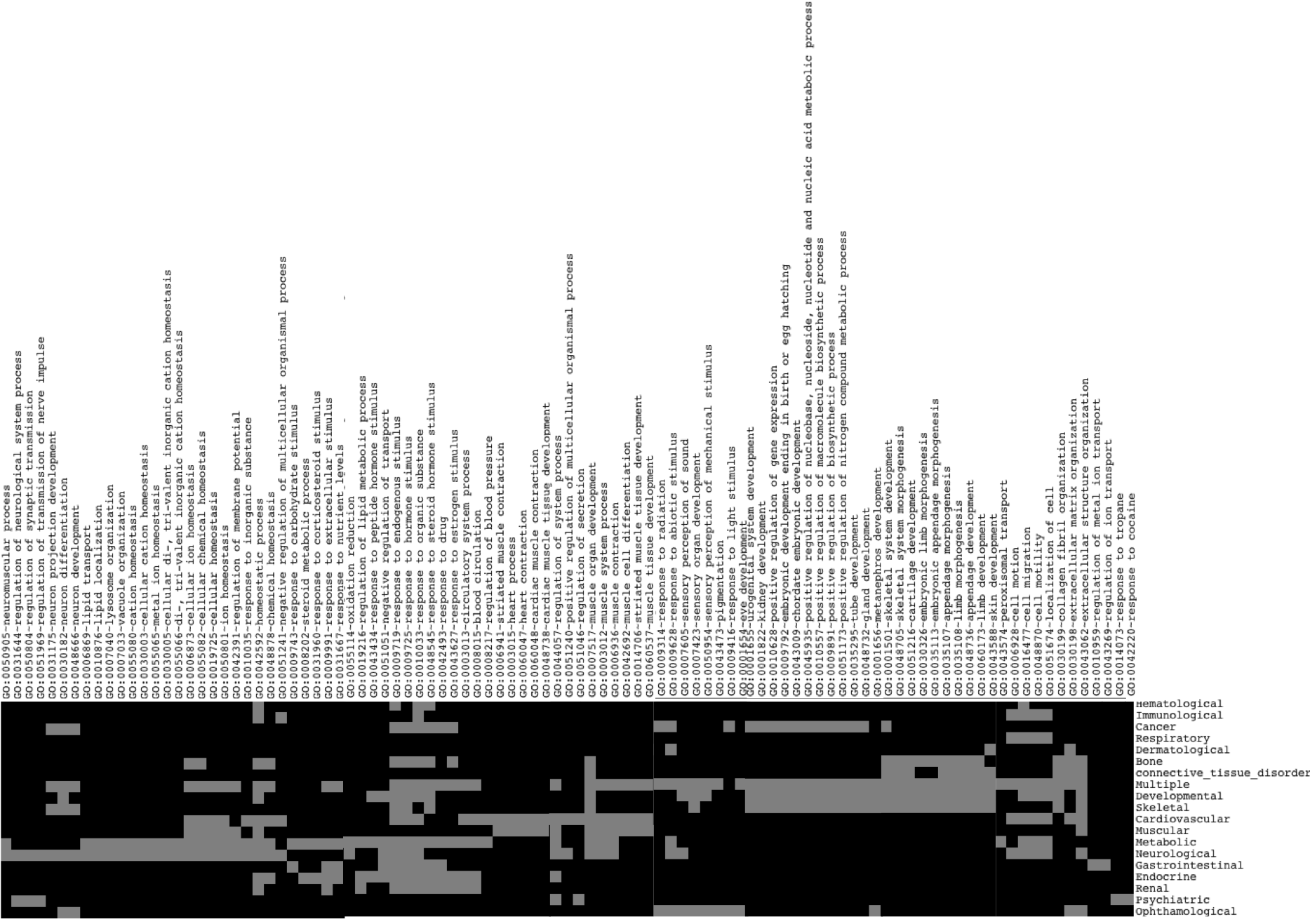

Supplement: S3 Fig — Association between GO biological processes and 19 human disease classes. The gray entries incidence a biological process and a disease class if the biological process is significantly enriched by the causative genes of the disease class. (TIFF) [file pone.0125138.s003.tiff]
