# Supplementary material for: Network-based Phenome-Genome Association Prediction by Bi-Random Walk
Source: PLoS One. 2015 May 1;10(5):e0125138. doi: 10.1371/journal.pone.0125138 (PMC4416812; doi:10.1371/journal.pone.0125138)
Supplement: S1 Table — CBG analysis with randomized phenotype-gene network are reported for comparison. (PDF) [file pone.0125138.s004.pdf]

Table S1. CBG statistics on human phenome-genome network (July 2014). CBG analysis with randomized phenotype-gene network are reported for comparison.

	Human phenotype-gene network		Randomized phenotype-gene network	
	Assoc #	Coverage	Avg. of Assoc #	Coverage
CBG1	689	34.68%	4.1	0.21%
CBG1 - 2	1232	62.00%	242.4	12.20%
CBG1 - 3	1743	87.72%	1407.8	70.85%
CBG1 - 4	1894	95.32%	1833.9	92.30%
CBG1 - 5	1915	96.38%	1882.8	94.76%
CBG1 - 6	1917	96.48%	1890.1	95.12%
CBG1 - 7	1922	96.73%	1893.2	95.28%
CBG1 - 8	1923	96.78%	1896.0	95.42%
CBG1 - 9	1924	96.83%	1899.0	95.57%
