# Supplementary material for: Network-based Phenome-Genome Association Prediction by Bi-Random Walk
Source: PLoS One. 2015 May 1;10(5):e0125138. doi: 10.1371/journal.pone.0125138 (PMC4416812; doi:10.1371/journal.pone.0125138)
Supplement: S2 Table — (A) This table reports the average AUCs across all the phenotypes by converged balanced BiRW(l = r) with different parameter α. The number of phenotypes with AUC more than 0.9 and 0.7 are also reported. The chosen α = 0.8 is among the best αs. (B) The tables report the AUC50, AUC100 and AUC300 by BiRW with the step parameter l and r (range from 1 to 5), where the parameter α is fixed as 0.8 based on the result in Fig 1A. The bold AUC scores are the best ones. Based on the results, the BiRW parameters are chosen as α = 0.8, l = 4 and r = 4 in 100-fold cross-validation. (PDF) [file pone.0125138.s005.pdf]

Table S2. Parameter tuning for BiRW with 100-fold cross-validation on OMIM May-2007.

(A) This table reports the average AUCs across all the phenotypes by converged balanced BiRW($l = r$) with different parameter α. The number of phenotypes with AUC more than 0.9 and 0.7 are also reported. The chosen $\alpha = 0.8$ is among the best αs.

α	0.1	0.2	0.3	0.4	0.5	0.6	0.7	0.8	0.9
AUC	0.741	0.760	0.764	0.773	0.776	**0.777**	**0.777**	**0.777**	0.775
≥0.9	527	590	613	626	626	625	625	**629**	624
≥0.7	711	758	770	789	795	801	800	797	791

(B) The tables report the AUC_{50}, AUC_{100} and AUC_{300} by BiRW with the step parameter l and r (range from 1 to 5), where the parameter α is fixed as 0.8 based on the result in Fig 1A. The bold AUC scores are the best ones. Based on the results, the BiRW parameters are chosen as α=0.8, l = 4 and r = 4 in 100-fold cross-validation.

B1. AUC_{50}

	r = 1	2	3	4	5
l=1	0.0066	0.0066	0.0066	0.0066	0.0066
2	0.3883	0.3883	0.1732	0.3307	0.1752
3	0.3872	0.4084	0.4123	0.2756	0.3151
4	0.3834	0.3926	0.4115	**0.4137**	0.2741
5	0.3807	0.3837	0.3938	0.4113	0.4113

B2. AUC_{100}

	r = 1	2	3	4	5
l=1	0.0118	0.0118	0.0118	0.0118	0.0118
2	0.4196	0.4196	0.2239	0.3815	0.2366
3	0.4179	0.4478	0.4562	0.3445	0.3680
4	0.4149	0.4277	0.4519	**0.4595**	0.3413
5	0.4122	0.4165	0.4317	0.4520	0.4578

B3. AUC_{300}

	r = 1	2	3	4	5
l=1	0.0383	0.0383	0.0383	0.0383	0.0383
2	0.4595	0.4595	0.3020	0.4719	0.3450
3	0.4584	0.5085	0.5173	0.4437	0.4637
4	0.4560	0.4813	0.5155	**0.5243**	0.4391
5	0.4535	0.4617	0.4874	0.5165	0.5241
