# Supplementary material for: Network-based Phenome-Genome Association Prediction by Bi-Random Walk
Source: PLoS One. 2015 May 1;10(5):e0125138. doi: 10.1371/journal.pone.0125138 (PMC4416812; doi:10.1371/journal.pone.0125138)
Supplement: S3 Table — This table reports the average AUCs across all phenotypes for PRINCE with the balance parameter α. (PDF) [file pone.0125138.s006.pdf]

Table S3. Parameter tuning for PRINCE in 100-fold cross-validation on OMIM May-2007. This table reports the average AUCs across all phenotypes for PRINCE with the balance parameter α.

	α	0.1	0.2	0.3	0.4	0.5	0.6	0.7	0.8	0.9
PRINCE	AUC	**0.751**	0.745	0.744	0.744	0.746	0.746	0.745	0.746	0.745
	$\geq$0.9	568	570	567	569	574	573	574	576	577
	$\geq$0.7	735	734	735	735	732	730	731	732	737
