# Supplementary material for: Network-based Phenome-Genome Association Prediction by Bi-Random Walk
Source: PLoS One. 2015 May 1;10(5):e0125138. doi: 10.1371/journal.pone.0125138 (PMC4416812; doi:10.1371/journal.pone.0125138)
Supplement: S4 Table — The table reports the average AUCs across all phenotypes for CIPHER SP (shortest path in PPI) and CIPHER DN (direct neighbor). (PDF) [file pone.0125138.s007.pdf]

**Table S4. Performance of CIPHER by 100-fold cross-validation on OMIM May-2007.** The table reports the average AUCs across all phenotypes for CIPHER SP (shortest path in PPI) and CIPHER DN (direct neighbor).

| | SP | DN |
|---|---|---|
| AUC | 0.494 | 0.602 |
| $\geq$0.9 | 116 | 161 |
| $\geq$0.7 | 342 | 459 |
