# Supplementary material for: Network-based Phenome-Genome Association Prediction by Bi-Random Walk
Source: PLoS One. 2015 May 1;10(5):e0125138. doi: 10.1371/journal.pone.0125138 (PMC4416812; doi:10.1371/journal.pone.0125138)
Supplement: S5 Table — The table reports a comparison of the ranking results by BiRW and four other methods, PRINCE, RWRH, CIPHER SP and CIPHER DN. The parameters α, l and r of BiRW are set by the experimental results in 100-fold cross-validation. AUCs up to 50, 100, 300, 500, 1000 and all false positives are reported. (PDF) [file pone.0125138.s008.pdf]

Table S5. AUCs of the cross-validation on OMIM May-2007. The table reports a comparison of the ranking results by BiRW and four other methods, PRINCE, RWRH, CIPHER SP and CIPHER DN. The parameters α, l and r of BiRW are set by the experimental results in 100-fold cross-validation. AUCs up to 50, 100, 300, 500, 1000 and all false positives are reported.

	AUC_{50}	AUC_{100}	AUC_{300}	AUC_{500}	AUC_{1000}	AUC
BiRW(0.8,4,4)	0.4137	0.4595	0.5243	0.5512	0.5897	0.8063
PRINCE(0.1)	0.4008	0.4353	0.4842	0.5036	0.5368	0.7909
RWRH(0.5,0.7,0.5)	0.2676	0.3056	0.3684	0.4002	0.4503	0.7475
CIPHER SP	0.0048	0.0082	0.0236	0.0364	0.0666	0.4942
CIPHER DN	0.0058	0.0093	0.0317	0.0479	0.0859	0.6017
