# Supplementary material for: Network-based Phenome-Genome Association Prediction by Bi-Random Walk
Source: PLoS One. 2015 May 1;10(5):e0125138. doi: 10.1371/journal.pone.0125138 (PMC4416812; doi:10.1371/journal.pone.0125138)
Supplement: S6 Table — The table reports a comparison of the ranking results by BiRW and 4 baselines, PRINCE, RWRH, CIPHER SP and CIPHER DN. The parameters α, l and r of BiRW are set by the experimental results in 100-fold cross-validation. AUCs up to 50, 100, 300, 500, 1000 and all false positives are reported. (PDF) [file pone.0125138.s009.pdf]

Table S6. AUCs of the prediction of the new disease genes in OMIM July-2014. The table reports a comparison of the ranking results by BiRW and 4 baselines, PRINCE, RWRH, CIPHER SP and CIPHER DN. The parameters α, l and r of BiRW are set by the experimental results in 100-fold cross-validation. AUCs up to 50, 100, 300, 500, 1000 and all false positives are reported.

	AUC_{50}	AUC_{100}	AUC_{300}	AUC_{500}	AUC_{1000}	AUC
BiRW(0.8,4,4)	0.1736	0.2115	0.2741	0.3077	0.3687	0.7369
PRINCE(0.1)	0.1439	0.1753	0.2310	0.2647	0.3227	0.7244
RWRH(0.5, 0.7, 0.5)	0.1599	0.1936	0.2611	0.3069	0.3849	0.7667
CIPHER SP	0.1068	0.1390	0.2165	0.2587	0.3233	0.7039
CIPHER DN	0.0935	0.1271	0.1838	0.2191	0.2754	0.6683
