# Supplementary material for: Network-based Phenome-Genome Association Prediction by Bi-Random Walk
Source: PLoS One. 2015 May 1;10(5):e0125138. doi: 10.1371/journal.pone.0125138 (PMC4416812; doi:10.1371/journal.pone.0125138)
Supplement: S7 Table — (PDF) [file pone.0125138.s010.pdf]

Table S7. A pairwise comparison by paired t-test of the ranking results in 100-fold cross-validation based on AUCs.

(A) p-values of comparing AUC_{50}

	BiRW(0.8,4,4)	PRINCE(0.1)	RWRH(0.5,0.7,0.5)	CIPHER SP	CIPHER DN
BiRW	NaN				
PRINCE	0.015	NaN			
RWRH	2.12e-37	8.74e-29	NaN		
CIPHER SP	5.41e-152	3.80e-144	9.24e-088	NaN	
CIPHER DN	1.34e-152	1.56e-144	3.08e-087	0.703	NaN

(B) p-values of comparing AUC_{100}

	BiRW(0.8,4,4)	PRINCE(0.1)	RWRH(0.5,0.7,0.5)	CIPHER SP	CIPHER DN
BiRW	NaN				
PRINCE	7.94e-05	NaN			
RWRH	5.17e-38	3.90e-25	NaN		
CIPHER SP	5.64e-170	5.11e-155	4.15e-98	NaN	
CIPHER DN	3.53e-171	1.73e-156	7.37e-98	0.688	NaN
