# Supplementary material for: Network-based Phenome-Genome Association Prediction by Bi-Random Walk
Source: PLoS One. 2015 May 1;10(5):e0125138. doi: 10.1371/journal.pone.0125138 (PMC4416812; doi:10.1371/journal.pone.0125138)
Supplement: S8 Table — (PDF) [file pone.0125138.s011.pdf]

Table S8. A pairwise comparison by paired t-test of the ranking results in test based on AUCs.

(A) p-values of comparing AUC_{50}

	BiRW(0.8,4,4)	PRINCE(0.1)	RWRH(0.5,0.7,0.5)	CIPHER SP	CIPHER DN
BiRW	NaN				
PRINCE	1.56E-05	NaN			
RWRH	0.0131	0.9683	NaN		
CIPHER SP	4.11E-11	0.0003	2.76E-08	NaN	
CIPHER DN	3.93E-11	5.49E-05	2.72E-09	0.0613	NaN

(B) p-values of comparing AUC_{100}

	BiRW(0.8,4,4)	PRINCE(0.1)	RWRH(0.5,0.7,0.5)	CIPHER SP	CIPHER DN
BiRW	NaN				
PRINCE	1.27E-07	NaN			
RWRH	0.0023	0.9836	NaN		
CIPHER SP	7.61E-12	0.0004	3.98E-08	NaN	
CIPHER DN	3.99E-11	1.58E-04	2.43E-08	0.1087	NaN
