# Supplementary material for: Network-based Phenome-Genome Association Prediction by Bi-Random Walk
Source: PLoS One. 2015 May 1;10(5):e0125138. doi: 10.1371/journal.pone.0125138 (PMC4416812; doi:10.1371/journal.pone.0125138)
Supplement: S9 Table — The parameters α, l and r of BiRW are tuned, and the best AUCs(up to 50, 100, 300, 500, 1000 and all false positives) with parameter α are reported. Based on the AUC50 results, the BiRW parameters for mouse are chosen as α = 0.7, l = 5 and r = 1 in 100-fold cross-validation (PDF) [file pone.0125138.s012.pdf]

Table S9. Performance of BiRW with 100-fold cross-validation on mouse phenome-genome network. The parameters α, l and r of BiRW are tuned, and the best AUCs(up to 50, 100, 300, 500, 1000 and all false positives) with parameter α are reported. Based on the AUC_{50} results, the BiRW parameters for mouse are chosen as α=0.7, l=5 and r=1 in 100-fold cross-validation

(A) AUC_{50} (α = 0.7)

	r = 1	2	3	4	5
l=1	0.0048	0.0048	0.0048	0.0048	0.0048
2	0.1397	0.1397	0.0175	0.0211	0.0179
3	0.1415	0.1298	0.1137	0.0190	0.0205
4	0.1416	0.1395	0.1198	0.0887	0.0207
5	**0.1419**	0.1419	0.1330	0.1087	0.0732

(B) AUC_{100} (α = 0.9)

	r = 1	2	3	4	5
l=1	0.0129	0.0129	0.0129	0.0129	0.0129
2	0.2257	0.2257	0.0367	0.0405	0.0333
3	0.2284	0.2146	0.1844	0.0383	0.0366
4	0.2293	0.2236	0.1905	0.1443	0.0363
5	**0.2297**	0.2270	0.2133	0.1699	0.1138

(C) AUC_{300} (α = 0.9)

	r = 1	2	3	4	5
l=1	0.0354	0.0354	0.0354	0.0354	0.0354
2	0.4395	0.4395	0.0880	0.0981	0.0699
3	0.4422	0.4200	0.3839	0.0976	0.0938
4	0.4431	0.4339	0.3904	0.3396	0.0981
5	**0.4434**	0.4382	0.4180	0.3670	0.3028

(D) AUC_{500} (α = 0.9)

	r = 1	2	3	4	5
l=1	0.0535	0.0535	0.0535	0.0535	0.0535
2	0.5658	0.5658	0.1243	0.1439	0.1074
3	0.5677	0.5457	0.5128	0.1394	0.1356
4	0.5684	0.5596	0.5193	0.4655	0.1406
5	**0.5685**	0.5642	0.5438	0.4959	0.4224

(E) AUC$_{1000}$ (α = 0.9)

	r = 1	2	3	4	5
l=1	0.0926	0.0926	0.0926	0.0926	0.0926
2	0.7602	0.7602	0.1988	0.2412	0.1744
3	0.7614	0.7345	0.7011	0.2189	0.2224
4	0.7616	0.7493	0.7069	0.6548	0.2191
5	**0.7617**	0.7549	0.7325	0.6820	0.6156

(F) AUC (α = 0.9)

	r = 1	2	3	4	5
l=1	0.6337	0.6337	0.6337	0.6337	0.6337
2	0.9688	0.9688	0.7084	0.7282	0.6996
3	0.9689	0.9653	0.9607	0.7277	0.7174
4	0.9689	0.9672	0.9613	0.9539	0.7213
5	**0.9690**	0.9680	0.9649	0.9579	0.9479
