# Supplementary material for: Network-based Phenome-Genome Association Prediction by Bi-Random Walk
Source: PLoS One. 2015 May 1;10(5):e0125138. doi: 10.1371/journal.pone.0125138 (PMC4416812; doi:10.1371/journal.pone.0125138)
Supplement: S10 Table — Parameter α ranges from 0.01 to 0.9 for tuning better. AUCs up to 50, 100, 300, 500, 1000 and all false positives are reported. (PDF) [file pone.0125138.s013.pdf]

Table S10. AUCs of PRINCE in 100-fold cross-validation on mouse phenome-genome network. Parameter α ranges from 0.01 to 0.9 for tuning better. AUCs up to 50, 100, 300, 500, 1000 and all false positives are reported.

	AUC_{50}	AUC_{100}	AUC_{300}	AUC_{500}	AUC_{1000}	AUC
0.01	0.0670	0.1245	0.3183	0.4633	0.7014	0.9612
0.05	0.0671	0.1248	0.3184	0.4640	0.7019	0.9615
0.1	0.0684	0.1257	0.3183	0.4640	0.7018	0.9615
0.2	0.0670	0.1242	0.3174	0.4634	0.7015	0.9615
0.3	0.0658	0.1230	0.3165	0.4625	0.7009	0.9614
0.4	0.0651	0.1223	0.3156	0.4616	0.7001	0.9613
0.5	0.0651	0.1216	0.3145	0.4606	0.6989	0.9611
0.6	0.0640	0.1195	0.3125	0.4585	0.6967	0.9609
0.7	0.0626	0.1177	0.3115	0.4569	0.6934	0.9605
0.8	0.0612	0.1154	0.3090	0.4539	0.6870	0.9596
0.9	0.0594	0.1125	0.3071	0.4495	0.6728	0.9576
