# Supplementary material for: Network-based Phenome-Genome Association Prediction by Bi-Random Walk
Source: PLoS One. 2015 May 1;10(5):e0125138. doi: 10.1371/journal.pone.0125138 (PMC4416812; doi:10.1371/journal.pone.0125138)
Supplement: S11 Table — CBG analysis with randomized phenotype-gene network are reported for comparison. (PDF) [file pone.0125138.s014.pdf]

Table S11. CBG statistics on mouse phenome-genome network. CBG analysis with randomized phenotype-gene network are reported for comparison.

	Mouse phenotype-gene network		Randomized phenotype-gene network	
	Assoc #	Coverage	Avg. of Assoc #	Coverage
CBG1	2497	25.48%	310.2	3.17%
CBG1 - 2	5038	51.42%	1001.1	10.22%
CBG1 - 3	6434	65.67%	1999.7	20.41%
CBG1 - 4	6993	71.37%	2666.2	27.21%
CBG1 - 5	7252	74.02%	2919.6	29.80%
CBG1 - 6	7462	76.16%	3002.1	30.64%
CBG1 - 7	7613	77.70%	3030.5	30.93%
CBG1 - 8	7704	78.63%	3037.9	31.01%
CBG1 - 9	7818	79.79%	3038.8	31.01%
